# The perceptions and experiences of caregivers of patients with dysphagia: A qualitative meta‐synthesis

**DOI:** 10.1002/nop2.2223

**Published:** 2024-09-01

**Authors:** QiaoLi Yi, LiYe Mao, WenYao Li, Fan Shen, ZongFeng Liao, HaiShan Huang, Ling Li

**Affiliations:** ^1^ Neurology Department, Tongji Hospital, Tongji Medical College Huazhong University of Science and Technology Wuhan China; ^2^ School of Nursing, Tongji Medical College Huazhong University of Science and Technology Wuhan China

**Keywords:** caregiver, dysphagia, meta‐synthesis, qualitative research

## Abstract

**Aims:**

To understand the perceptions and experiences of family caregivers of adult patients with dysphagia.

**Background:**

Dysphagia is a common symptom and burdens caregivers greatly. There is a growing body of studies concentrating on caregivers and caregiving experiences. However, no qualitative meta‐synthesis has been conducted to explore the perceptions and experiences of family caregivers.

**Design:**

A qualitative meta‐ethnography.

**Methods:**

A search was conducted for relevant articles in six electronic databases (PubMed, Web of Science, CINAHL, Ovid, Cochrane Library, ProQuest) and two Chinese databases (CNKI, Wanfang Data) from inception to February 2023. The Joanna Briggs Institute Qualitative Assessment and Review Instrument (JBI‐QARI) was used to evaluate study quality. The meta‐ethnographic method was used to synthesize data from qualitative studies. The study was reported according to EQUATOR guidelines.

**Results:**

Eleven studies were included and three themes emerged: (1) emotion and perception, (2) change and challenge (3) adaption and coping.

**Conclusion:**

This review highlighted the challenges and positive coping experienced by caregivers. Findings directly inform the development and implementation of supportive interventions to reduce caregivers' stress and promote adaptive coping.

**Relevance to clinical practice:**

Pay attention to the needs of family caregivers of dysphagia. Family caregivers' perceived severity of dysphagia requires assessment. Caregivers need knowledge, support, and guidance to reduce their burden and fulfill their role.

## 1 INTRODUCTION

Dysphagia can be defined as a ‘difficulty in oral preparation for the swallow or in moving a spoonful (bolus) from the mouth to the stomach’ (Andrade et al., 2018). Theoretically, dysphagia can occur at any stage of life, while it's more frequently present in patients with aging, neurodegenerative disease and/or head and neck diseases (Turley & Cohen, 2009). Dysphagia affects the afflicted patients and their caregivers as a symptom that is usually accompanied for a long time. Studies have reported that caregivers experience high disease burdens compounded by unmet nursing needs (Namasivayam‐MacDonald & Shune, 2018; Ninfa et al., 2021). It is necessary to explore and obtain the perceptions and experiences of caregivers. Qualitative meta‐synthesis can overcome the inadequacy of single qualitative studies and be used to understand perceptions and experiences more deeply (LeSeure & Chongkham‐Ang, 2015). Understanding and exploring caring experiences can guide healthcare providers to develop targeted medical interventions to support this group.

## 2 BACKGROUND

Dysphagia refers to a subjective sensation of difficulty swallowing and there are a variety of etiologies (Abdel et al., 2015). The most common conditions leading to dysphagia include stroke, head and neck cancer, or progressive neurologic disease (Christmas & Rogus‐Pulia, 2019). A large–scale study in the United States found that dysphagia affects 16.1% of adults at some point during their lives (Adkins et al., 2020).

Dysphagia can lead to many serious consequences, including pneumonia, malnutrition, dehydration and death. It is also associated with increased psycho‐emotional and economic burden, decreased quality of life and social isolation (Lin & Shune, 2020; Stoner et al., 2019; Jones et al., 2018; Patel et al., 2018). A diagnosis of dysphagia impacts not only patients but also their family caregivers. Since the patients are often frail and have alimentary restrictions, a caregiver usually prepares the meals, ensures a relatively stable weight and supervises eating in case of choking (Rabiei et al., 2020). In addition to disruption in lifestyle and additional labor, caregivers are prone to suffering negative emotions such as sadness, helplessness and depression (Namasivayam‐MacDonald & Shune, 2018; Rangira et al., 2022). A recent systematic review revealed 71% of caregivers of adults with dysphagia experience different levels of burden (Rangira et al., 2022).

Caregivers and caregiving experience are gradually gaining attention in recent years. Experience is subjective and a qualitative study is ideal for a thorough understanding of experience. Compared to quantitative studies, qualitative research can offer essential insights and describe the nature of the problem. Studies that attempted to quantify the effects of caregiving on caregivers were hard to resoundingly provide empirical comprehension of the nature or essence of the caregiving experience (LeSeure & Chongkham‐Ang, 2015). Some qualitative studies have attempted to explore the caregivers' experiences of patients with dysphagia to understand the feelings, needs and caregiving concerns of caregivers (Howells et al., 2021; Lisiecka et al., 2020; Robinson et al., 2022). Yet these results are isolated from each other. Due to variations in race, situation, research methods and so on, a single qualitative study is insufficient for understanding the phenomenon.

A qualitative synthesis is defined as ‘any methodology whereby study findings are systematically interpreted through a series of expert judgements to represent the meaning of the collected work (Bearman & Dawson, 2013) it provides insight into the experience of the caregivers of patients with dysphagia and their responses. Qualitative synthesis aims to aggregate and synthesize existing qualitative evidence to comprehensively explore phenomena in order to build a more in‐depth understanding than a single qualitative study (LeSeure & Chongkham‐Ang, 2015). The qualitative synthesis methods conclude meta‐ethnography method, thematic synthesis method, textual narrative synthesis method, et al. Meta‐ethnography is a commonly used method of qualitative synthesis approach in healthcare research (Sattar et al., 2021), which is an inductive, interpretative approach. Meta‐ethnography enables researchers to systematically compare a set of studies focused on the research question and achieve a new interpretation of the selected studies rather than simply integrating and describing studies (Saragosa et al., 2022).

To our knowledge, no meta‐ethnography of caregivers' experiences of adult patients with dysphagia exists. This review aims to get a comprehensive picture of the caregiver experience, including the caregiver's feelings, perceptions, behaviours, difficulties, coping strategies, support needed, etc. Specifically, we asked the following research questions: (1) what is the caregiver's perception of dysphagia? (2)What challenges do caregivers face when caring for adult patients with dysphagia? (3) What is the adaption process like? The findings can guide clinical practice and policy development to improve adult patients' and caregivers' physical and psychological health and improve dysphagia‐related quality of life.

## 3 METHODS

### 3.1 Search strategy

Searches were performed in both English and Chinese databases (PubMed, Web of Science, CINAHL, Ovid, Cochrane Library, ProQuest, China National Knowledge Infrastructure [CNKI] and Wanfang Data) from inception to February 2023. Additionally, we checked the reference lists of discovered papers for additional papers that might be candidates. We combined controlled vocabulary (MeSH terms) with free text terms for our search strategy (Appendix S1).

### 3.2 Eligibility criteria

The eligibility criteria will follow the PICOS design (Participant‐Interest in phenomena‐Context‐Study). Studies were included if they covered a sample of adults with dysphagia and explored their caregivers' perceptions and/or caring experiences of dysphagia. The inclusion of studies without full texts, duplicate publications, quantitative studies, letters and reviews was excluded (Table 1).

**TABLE 1:** inclusion and exclusion criteria.

Inclusion criteria	Exclusion criteria
1. Participants (P): Informal caregivers of people with swallowing disorders.	1. Quantitative studies, conference abstracts, case report, and protocol, systematic reviews and other types of reviews, papers with incomplete data, duplicate records
2. Interest of phenomena (I): Caregiver's perceptions, feelings and nursing experience.	2. For the mixed‐method studies, the qualitative results cannot be extracted.
3. Context (Co): Patients at the hospital or home or healthcare facilities.	3. The experiences of caregivers in qualitative studies cannot be extracted independently
4. Study (S): Qualitative research or mixed research containing qualitative methods, the methodology includes phenomenology, grounded theory, ethnography and other qualitative research methods.	
5. When caregivers and others were recruited, the studies were included only if the researchers presented data pertaining to caregivers' experiences separate from others' experiences	

### 3.3 Quality appraisal

The Joanna Briggs Institute Qualitative Assessment and Review Instrument (2022) was used to appraise the quality of each included study (QARI). The criteria covered 10 questions and each question has four choices: yes, no, unclear or not applicable. Studies were classified according to whether they met (Grade A), partially met (Grade B) or did not meet the quality criteria at all (Grade C). Studies rated as grade C were excluded. Two reviewers independently conducted the assessment and disagreements were discussed with a third researcher until a consensus was reached.

### 3.4 Data extraction

The following data were gathered: Year published; Author name(s); Title; Number of participants; Research type; Data collection and analysis method; Aetiology of dysphagia. Meanwhile, information on caregivers' experiences and perceptions was extracted. Two reviewers independently extracted data from the included studies to ensure the validity of the data extraction and any disagreement would be discussed with a third reviewer.

### 3.5 Data synthesis

We followed seven steps for meta‐ethnography: (a) getting started by identifying phenomena of interest, (b) decision on related studies, (c) to read included studies, (d) determining how the studies are related, (e) translating the studies into one another, (f) synthesizing translations and (g) expressing the synthesis (France et al., 2019).The extracted data were summarized into concepts and these concepts were compared to create a list of key concepts. The notion of first‐order, second‐order and third‐order constructs are typically used in meta‐ethnography (Britten et al., 2002). Traditionally, first‐order constructs represent the original data of each paper. Second‐order constructs are the interpretations of primary data made by authors, and third‐order constructs are the reviewers' interpretations. While the first‐ and second‐order constructs were indistinguishable sometimes. We will draw from the method in the previous study (Kim et al., 2020). The participant's and the author's interpretations would be conceptualized as first‐order constructs in this study. Then, the conceptualized themes were compared and contrasted to formulate second‐order constructs. Finally, third‐order constructs would be formed through further abstracting and generalizing these second‐order constructs. The whole research team reviewed the findings, confirmed relevance to the original study and discussed how to express the synthesis. And the PRISMA (Preferred Reporting Items for Systematic Reviews) 2020 checklist was used to report the meta‐synthesis (2022a) (Appendix S2).

## 4 FINDINGS

### 4.1 Search outcomes

**FIGURE 1:**
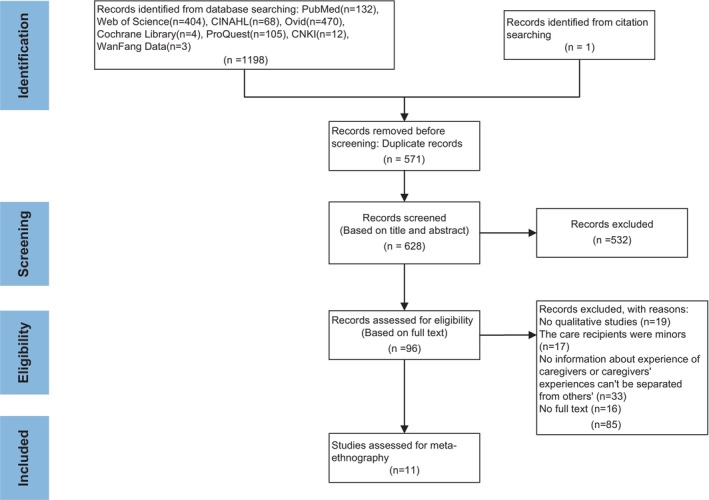
Flow grams of studies selection.

The initial search retrieved 1198 studies; we imported all search results into EndNote software and 570 duplicates were removed, 532 were excluded after checking titles and abstracts because they were considered irrelevant. Two reviewers read the remaining full articles to identify which would be included based on the inclusion criteria. When there were disagreements between the two researchers, a third researcher was consulted to determine the final eligibility of the articles. Finally, a total of 11 articles were included in this meta‐analysis. The PRISMA (preferred reporting items for systematic reviews) flow diagram (Figure 1) is shown below.

### 4.2 Characteristics of the included studies

This meta‐synthesis of qualitative studies included 11 studies from six countries: Ireland (*n* = 1) (Lisiecka et al., 2020), Australia (*n* = 2) (Howells et al., 2021; Nund et al., 2014), China (*n* = 3) (Fan et al., 2022; Liu et al., 2021; Ren et al., 2019), UK (*n* = 1) (Robinson et al., 2022), Sweden (*n* = 1) (Johansson & Johansson, 2009), Canada (*n* = 3) (LaDonna et al., 2016; Penner et al., 2012; Smith et al., 2015). Detailed information about these studies is revealed in Table 2.

**TABLE 2:** Characteristic of included studies.

Author	Country	Demographics (*n*, male/female)	Aims	Research design	Data collection	Data analysis	Aetiology of dysphagia
(Lisiecka et al., 2020)	Ireland	*N* = 10 (2/8)	To investigate the experiences of dysphagia from the perspective of family caregivers of people diagnosed with amyotrophic lateral sclerosis	Interpretative phenomenological	Individual interviews and optional mealtime preparation observations	The six steps of Interpretative phenomenological analysis(IPA)	Amyotrophic lateral sclerosis
(Howells et al., 2021)	Australia	*N* = 15 (2/13)	To understand the experience of supporting a person with dysphagia of varying etiologies in the community from the caregiver perspective	A qualitative descriptive approach grounded in phenomenology	Face‐to‐face, individual semi‐structured interviews	Thematic analysis	Stroke (*n* = 5) Parkinson's disease(*n* = 7) General aging (*n* = 1) Arteriovenous malformation (AVM) (*n* = 1) Base of tongue (BOT) cancer (*n* = 1)
(Robinson et al., 2022)	UK	*N* = 5 (2/3)	To explore family members' experiences of living with a spouse with post‐stroke dysphagia	An exploratory qualitative methodology	Semi‐structured interviews	Thematic analysis	Stroke
(Fan et al., 2022)	China	*N* = 10 (3/7)	To explore the experience and needs of stroke caregivers for dysphagia care management	Phenomenological	Semi‐structured interviews	Colaizzi's phenomenological analysis method	Stroke
(Ren et al., 2019)	China	*N* = 10 (3/7)	To understand family members' difficult and needs of caring older patients with dysphagia	Phenomenological	Semi‐structured interviews	Colaizzi's phenomenological analysis method	Unspecified
(Liu et al., 2021)	China	*N* = 11 (5/6)	To understand the needs of caregivers of stroke patients with dysphagia	Phenomenological	Semi‐structured interviews	Colaizzi's phenomenological analysis method	Stroke
(Nund et al., 2014)	Australia	12 (2/10)	To report on the experiences of carers of people with dysphagia (non‐gastrostomy dependent) following nonsurgical treatment for HNC and to identify the support needs of this group	A qualitative descriptive approach based on phenomenology	An in‐depth, semi‐structured, individual interview	Thematic analysis	Head and neck cancer (HNC)
(Johansson & Johansson, 2009)	Sweden	9 (1/8)	To describe relatives' experiences of their next of kin's eating and swallowing disorders	A qualitative descriptive approach	Individual interview	A constant comparative approach	Stroke or traumatic injuries
(Smith et al., 2015)	Canada	14 (4/10)	To understand primary caregivers'(PCGs)’ beliefs, values, and responses to dysphagia and dietary modifications in the palliative care setting	A qualitative descriptiveapproach	Semi‐structured interviews/observations	A thematic analysis based on a descriptive approach	Unspecified
(Penner et al., 2012)	Canada	6 (2/4)	To explicate the lived experience of caring for a dysphagic relative with advanced head and neck cancer receiving tube feeding	A Descriptive Phenomenological Study	Interviews (twice)	Spiegelberg's three‐step process of intuiting, phenomenological analysing, and phenomenological describing	Head and neck cancer (HNC)
(LaDonna et al., 2016)	Canada	6 (4/2)	To explore the experiences of caregivers living with those with DM1and dysphagia	An interpretive phenomenological approach	Semi‐structured interviews	van Manen's suggested steps for phenomenological analysis	Myotonic Dystrophy(DM1)

### 4.3 Quality appraisal of the included studies

Appraisal of the 11 studies with the JBI (Joanna Briggs Institute) Critical Appraisal Checklist revealed the overall quality of the body of evidence to be moderate to good. On the basis of this assessment, no study was excluded; full results are listed in Table 3.

**TABLE 3:** Methodological quality of the included studies.

Author	Item1	Item2	Item3	Item4	Item5	Item6	Item7	Item8	Item9	Item10	Overall Appraisal
(Lisiecka et al., 2020)	Y	Y	Y	Y	Y	Y	Y	Y	Y	Y	A
(Howells et al., 2021)	Y	Y	Y	Y	Y	N	Y	Y	Y	Y	B
(Robinson et al., 2022)	Y	Y	Y	Y	Y	Y	Y	Y	Y	Y	A
(Fan et al., 2022)	U	Y	Y	Y	Y	N	Y	Y	Y	Y	B
(Ren et al., 2019)	U	Y	Y	Y	Y	N	N	Y	U	Y	B
(Liu et al., 2021)	U	Y	Y	Y	Y	Y	Y	Y	U	Y	B
(Nund et al., 2014)	Y	Y	Y	Y	Y	N	N	Y	Y	Y	B
(Johansson & Johansson, 2009)	U	Y	Y	Y	Y	N	N	Y	Y	Y	B
(Smith et al., 2015)	U	Y	Y	Y	Y	N	N	Y	Y	Y	B
(Penner et al., 2012)	Y	Y	Y	Y	Y	N	Y	Y	Y	Y	B
(LaDonna et al., 2016)	Y	Y	Y	Y	Y	N	N	Y	Y	Y	B

*Note*: 1. Is there congruity between the stated philosophical perspective and the research methodology? 2.Is there congruity between the research methodology and the research questions or objectives? 3.Is there congruity between the research methodology and the data collection methods? 4.Is there congruity between the research methodology and the representation and the data analysis? 5.Is there congruity between the research methodology and the interpretation of results? 6.Is there a statement locating the researcher culturally or theoretically? 7.Is the influence of the researcher on the research, and vice‐ versa, addressed? 8. Are participants and their voices adequately represented? 9. Is the research ethical according to current criteria or, for recent studies, and is there evidence of ethical approval by an appropriate body? 10.Do the conclusions drawn from the research report flow from the analysis or interpretation of the data?

Abbreviations: NA, not applicable; N, no; U, unclear; Y, yes.

### 4.4 Main findings of the meta‐synthesis

Three themes were revealed from caregivers' caring experiences and perceptions: (1) emotion and perception, (2) change and challenge and (3) adaption and coping. Table 4 displays synthesized themes and subthemes associated with first‐, second‐ and third‐order constructs.

**TABLE 4:** caregivers' perceptions and experiences of caring for patients with dysphagia.

Third order constructs (theme)	Second‐order constructs (sub‐theme)	Key concepts from first‐order constructs
Emotion and perception	Emotional experience	Frustration associated with dysphagia (Howells et al., 2021; LaDonna et al., 2016; Lisiecka et al., 2020; Nund et al., 2014; Ren et al., 2019; Smith et al., 2015) Feel anxious and stressed for being a caregiver (Fan et al., 2022; Howells et al., 2021; Lisiecka et al., 2020; Liu et al., 2021; Robinson et al., 2022) feel empathy and guilty for patients (Johansson & Johansson, 2009; Lisiecka et al., 2020; Nund et al., 2014; Robinson et al., 2022) feel embarrassed and discomfort for the change in the patient's body image (Howells et al., 2021; Johansson & Johansson, 2009) Caregiver burnout (LaDonna et al., 2016; Lisiecka et al., 2020; Penner et al., 2012)
	Cognitive experience	The awareness of caregiver capacity (Howells et al., 2021; LaDonna et al., 2016; Smith et al., 2015) The awareness of dysphagia (Fan et al., 2022; Howells et al., 2021; LaDonna et al., 2016; Lisiecka et al., 2020; Robinson et al., 2022; Smith et al., 2015) The awareness of feeding (Penner et al., 2012; Smith et al., 2015)
Change and challenge	Dining dilemma	More time and effort required for eating (Johansson & Johansson, 2009; Nund et al., 2014) The stressful dining atmosphere (Johansson & Johansson, 2009; Lisiecka et al., 2020; Nund et al., 2014; Penner et al., 2012) Eating outside is a challenge (Howells et al., 2021; Johansson & Johansson, 2009; Nund et al., 2014; Penner et al., 2012)
	Affected personal life	Be alert every moment (Howells et al., 2021; Lisiecka et al., 2020). Take up personal time (Johansson & Johansson, 2009; Lisiecka et al., 2020) Physical health is affected (Johansson & Johansson, 2009; LaDonna et al., 2016; Lisiecka et al., 2020). Social limitations (Fan et al., 2022; LaDonna et al., 2016; Penner et al., 2012) High financial stress (Fan et al., 2022; Liu et al., 2021; Ren et al., 2019)
	Altered family relationships	The colder and worse relationships (Howells et al., 2021; LaDonna et al., 2016; Lisiecka et al., 2020; Nund et al., 2014; Robinson et al., 2022) The more harmonious and intimate relationships (Howells et al., 2021; Nund et al., 2014; Robinson et al., 2022)
	In need of knowledge and support	In need of professional education and formal support (Fan et al., 2022; Howells et al., 2021; Lisiecka et al., 2020; Liu et al., 2021; Ren et al., 2019) In need of support from a family member/friend or the informal support (Howells et al., 2021; Johansson & Johansson, 2009)
Adaption and coping	Strive to restructure life	Learn knowledge of dysphagia (Fan et al., 2022; Nund et al., 2014; Robinson et al., 2022) Develop coping strategies for caregiving (Howells et al., 2021; Johansson & Johansson, 2009; Nund et al., 2014; Robinson et al., 2022; Smith et al., 2015) Develop coping strategies for relieving stress (Howells et al., 2021; LaDonna et al., 2016; Lisiecka et al., 2020; Liu et al., 2021; Nund et al., 2014)
	Post‐traumatic growth	Understanding of self‐role and family relationships (LaDonna et al., 2016; Penner et al., 2012) Be active (Howells et al., 2021; Lisiecka et al., 2020; Penner et al., 2012) Get a sense of accomplishment and fulfilment (Fan et al., 2022; Penner et al., 2012)

#### 4.4.1 Emotion and perception

The theme describes caregivers' emotional and cognitive experiences related to patients' dysphagia, including two subthemes: emotional experience and cognitive experience.

##### Emotional experience

- Adult patients suffering from swallowing impairments play as a traumatic accident for caregivers, which generates complicated and intense emotional responses among carers. Frustration was reported most as they realized that patients' health was declining (Howells et al., 2021; LaDonna et al., 2016; Lisiecka et al., 2020; Nund et al., 2014; Ren et al., 2019; Smith et al., 2015). Feeling anxious and stressed may follow, caregivers worried about the patient's progress and prognosis and get caught up in fear of being unequal to a caring job (Fan et al., 2022; Howells et al., 2021; Lisiecka et al., 2020; Liu et al., 2021; Robinson et al., 2022). Observing the patients unable to eat properly made some caregivers feel commiserative and guilty (Johansson & Johansson, 2009; Lisiecka et al., 2020; Nund et al., 2014; Robinson et al., 2022), while it also gave rise to embarrassment and discomfort in some caregivers (Howells et al., 2021; Johansson & Johansson, 2009). Some caregivers experienced large burdens in caring for their relatives; they expressed to be exhausted due to a caregiver role (LaDonna et al., 2016; Lisiecka et al., 2020; Penner et al., 2012).

She's [wife] sort of not able to eat […]. I feel very bad about that. (Lisiecka et al., 2020).

The frightening part is I don't know where this [dysphagia] is going to lead (Howells et al., 2021).

##### Cognitive experience

Several studies contributed to this theme. In some studies, caregivers talked about the understanding of their nursing competence. Caregiver Capacity is influenced by individual factors, such as life experience, belief and education (Howells et al., 2021; LaDonna et al., 2016; Smith et al., 2015). Several studies also mentioned caregivers' understanding of dysphagia and the feeding process. In some caregivers' opinion, dysphagia was easy to identify; coughing, choking and prolonged meals were common symptoms (Lisiecka et al., 2020). Feeding was seen as an intervention to support life rather than a process of enjoyment or pleasure; the key point of feeding is to ensure adequate nutrition and patient safety (Penner et al., 2012; Smith et al., 2015). Another viewpoint supported by five studies was that dysphagia was not the only concern regarding patients and caregivers. Caregiving is complicated; dysphagia was neither the only nor the most severe problem in some patients (Fan et al., 2022; Howells et al., 2021; LaDonna et al., 2016; Robinson et al., 2022; Smith et al., 2015). Patients experienced a variety of symptoms. Simultaneous management of several symptoms is more likely to be a norm for caregivers.

Well, they [children] can see that [dysphagia] (Lisiecka et al., 2020). My role is to make sure that the food, you know, he was getting his nutrition (Penner et al., 2012)

I have more things that are harder [to manage than dysphagia] because he's got incontinence problems. (Howells et al., 2021).

#### 4.4.2 Change and challenge

The main theme, ‘change and challenge’, describes the practical changes and problems in caregivers' life after diagnosing patients' dysphagia. Four subthemes were identified to support this theme, dining dilemma, affected personal life, altered family relationships and in need of knowledge and support.

##### Dining dilemma

Several studies have pointed out that the most immediate change caused by dysphagia is a dining dilemma (Howells et al., 2021; Johansson & Johansson, 2009; Lisiecka et al., 2020; Nund et al., 2014; Penner et al., 2012). Due to limitations on the type and texture of food for patients, caregivers have to spend more time on food preparation or intake (Johansson & Johansson, 2009; Nund et al., 2014). And patients unable to eat naturally also caused a stressful dining atmosphere in the family. The feeling of guilt about eating in front of patients is one of them (Johansson & Johansson, 2009; Lisiecka et al., 2020; Penner et al., 2012); being wary and cautious to ensure patients' diet safety and nutrition intake is the other (Johansson & Johansson, 2009; Lisiecka et al., 2020; Nund et al., 2014). In addition, the challenge of eating outside is mentioned by four studies (Howells et al., 2021; Johansson & Johansson, 2009; Nund et al., 2014; Penner et al., 2012). Several causes for reducing dining outside are as follows. First, caregivers are frustrated by difficulty finding appropriate food for patients (Howells et al., 2021). Second, the caregivers may experience shame due to patients unable to eat normally being exposed to the public (Johansson & Johansson, 2009; Penner et al., 2012). Moreover, eating outside would potentially increase the workload of the caregiver (Howells et al., 2021; Nund et al., 2014).

Preparing food is more intense because you are always thinking more carefully about it (Nund et al., 2014).

Going out to dinner…it's better not to do it now because it's just too hard to find something that suits (Nund et al., 2014).

##### Affected personal life

- The caregivers focus on the patient's demands, which affects the caregiver's life in all aspects, physiological, psychological, social contact, occupation and so on. Caregivers reported that they need to be alert every moment to prevent patients' injury (Howells et al., 2021; Lisiecka et al., 2020). And the caring work takes over sleep time or playtime and finally affects their health (Johansson & Johansson, 2009; LaDonna et al., 2016; Lisiecka et al., 2020). They must reduce social activities and develop a detailed nursing plan to balance care tasks with work (Fan et al., 2022; LaDonna et al., 2016; Penner et al., 2012). Moreover, three studies in China reported that dysphagia imposes a financial burden on households (Fan et al., 2022; Liu et al., 2021; Ren et al., 2019).

The biggest thing is […] sleep deprivation (sighs) (Lisiecka et al., 2020).

You just sort of had things scheduled at specific times throughout the day. And, uh, my outside activities were sort of …between the feedings… (Penner et al., 2012).

##### Altered family relationships

The findings show that change in a family relationship is not necessarily negative. Some caregivers reported colder and worse relationships with the patients due to physical or mental burden (Howells et al., 2021; LaDonna et al., 2016; Lisiecka et al., 2020; Nund et al., 2014; Robinson et al., 2022). While to some extent, the caregiving task also induced family responsibility in some caregivers, who reported more harmonious and intimate family relationships after being in caring roles (Howells et al., 2021; Nund et al., 2014; Robinson et al., 2022).

But he was always the boss, and he's still the boss…Yeah he's no empathy at all from my side of the problem. (Lisiecka et al., 2020).

We make a good team; we're one. As long as we stick together, we'll be alright. (Howells et al., 2021).

##### In need of knowledge and support

Professional education and support about dysphagia were highly valued by caregivers (Howells et al., 2021). Several studies (Fan et al., 2022; Lisiecka et al., 2020; Liu et al., 2021; Ren et al., 2019) demonstrate that informal caregivers lack dysphagia‐related knowledge by varying degrees. Professional guidance was required to assist caregivers' caring process. Besides, a study from Australia (Nund et al., 2014) emphasized that the guidance provided needed to be practical and individualized, and the medical terms should be avoided in expression.

I suppose that [choking] is a fear in a way now, and the choking episode on my part, what do I do? Call an ambulance? What can they do? I don't know. (Lisiecka et al., 2020).

[Health professionals] think their knowledge is everybody's knowledge and it's not. They [have] got to use…patient language. (Nund et al., 2014).

In addition to support from the medical staff mentioned above, support from a family member/friend or informal support is also needed. (Howells et al., 2021; Johansson & Johansson, 2009). Caregivers could share caring responsibilities with other family members or get emotional support from relatives or friends.

It's very hard for one person to be doing all of the things… You need to have a network of us kind of working in together to get the work done. (Howells et al., 2021).

#### 4.4.3 Adaption and coping

This theme refers to corresponding behaviour and psychological adjustment accommodating to life changes. Caregivers make efforts to provide caregiving and improve quality of life. They also expressed psychological growth from caring experience. This theme includes two subthemes: strive to restructure life, post‐traumatic growth.

##### Strive to restructure life

The caregivers reported various behaviours reflecting caregivers' efforts to accommodate the demands of patients and themselves. Caregivers attempt to learn nursing knowledge of dysphagia (Fan et al., 2022; Nund et al., 2014; Robinson et al., 2022). And they develop their own coping strategies to assist patients, such as supporting meal (Howells et al., 2021; Johansson & Johansson, 2009; Nund et al., 2014; Robinson et al., 2022; Smith et al., 2015), oral cleaning (Fan et al., 2022; Johansson & Johansson, 2009), concentrating on patients' rehabilitation exercise (Nund et al., 2014; Ren et al., 2019) and giving psychological comfort (Fan et al., 2022; Johansson & Johansson, 2009). In addition, caregivers reported behaviours to relieve their caregiving burden, which includes seeking support from a family member/friend (Howells et al., 2021; Johansson & Johansson, 2009; Liu et al., 2021; Nund et al., 2014) and shifting attention through communication or participation in work (LaDonna et al., 2016; Lisiecka et al., 2020).

I put everything in through a syringe, and give him his medications and that, and it won't go, um, so I but I've learnt how to sort of um unblock that now (Robinson et al., 2022).

##### Post‐traumatic growth

Although caregivers reported drastic life changes and intense emotional responses, the caregivers still acknowledged the positive aspects of the caring experience. They tried to adopt caregiving roles and accept caregiving being part of their lives. Caregivers reported having a more insightful understanding of self‐role and family relationships (LaDonna et al., 2016; Penner et al., 2012) who are willing to actively facing reality (Howells et al., 2021; Lisiecka et al., 2020; Penner et al., 2012) and get a sense of accomplishment and fulfilment when provided satisfactory caregiving (Fan et al., 2022; Penner et al., 2012).

…the roles we're in right now are not roles we would choose to be in… (Penner et al., 2012).

I would say carry on as normal once you get over the initial shock and make the most of everyday and don't let anything pass by that you could do (Lisiecka et al., 2020).

## 5 DISCUSSION

This is the first meta‐ethnography to explore the perceptions and experiences of caregivers for adult patients with dysphagia; three overarching themes are identified that exceed the results of a single qualitative study, which would help healthcare workers develop effective strategies to assist adult patients and caregivers.

This meta‐synthesis reported a certain proportion of under‐perception of dysphagia among caregivers. Although included qualitative studies focused on swallowing disorders, these participants unconsciously referred to other symptoms (language barrier, limb function, incontinence, et al.) of the patients in their interviews. From their perspective, swallowing disorders were neither the only nor the most important symptom. Caregivers neglecting swallowing disorders probably represent that caregivers perceived severity of dysphagia is low. The Common‐Sense Model of Self‐Regulation (CSM) provides an important perspective that caregivers' perceived severity of dysphagia influences their emotional responses and behavioural choices (Leventhal et al., 1998). Dysphagia severity is relevant to caregiver burden (Suzuki et al., 2022). However, our results may indicate that caregivers' subjective perception of dysphagia severity may be the potential influencing factor of caregiver burden since it could impact caregivers' mood, cognition and behaviour. Thus, healthcare providers need to focus more on caregivers' subject perception. Additional research is needed to determine the differences between the actual severity of dysphagia and caregivers' perceived severity, which could guide healthcare providers to develop targeted interventions to enhance caregivers' understanding of the condition in healthcare interactions.

The first and second themes demonstrated that family caregivers experienced considerable lifestyle changes, and physical and emotional burdens. This is consistent with the previous results of quantitative research (Namasivayam‐MacDonald & Shune, 2018; Rangira et al., 2022). Our results differed in that adult patients with dysphagia often suffering from other symptoms could further heighten caregivers' burden. While caregivers receive some external support, it's still medically, educationally, or emotionally insufficient. Several potential reasons exist for inadequate support. First, dysphagia is generally recognized to decrease self‐esteem and social isolation (Farri et al., 2007). This disruption to social relationships probably causes inadequate support for caregivers and patients. Second, many healthcare professionals do not enough in the provision of information, training, instrumental and appraisal support, which could be caused by health professionals' inadequate knowledge of the management of dysphagia (Li et al., 2022) or overburdened with multiple clinic tasks (Gab et al., 2020). This meta‐synthesis indicates that caregivers need external support and services. Thus, it is necessary to put more medical resources and develop effective ways to reduce caregivers' psychosocial and physical burdens. The results indicate sources of caregiver stress include, but are not limited to, emotional and physical limits, social isolation or lack of knowledge or skill and caregiving burnout. This displays the diversity and individual variability of caregivers' needs, so comprehensive assessments should be performed first when developing support strategies. Secondly, it also means caregivers group with needs in multiple areas and calls for multidimensional support. The support or service should be provided by multi‐institutional and multidisciplinary teams. Finally, treating and managing dysphagia highly depends on the cause (McCarty & Chao, 2021). Which suggests that medical staff should consider the actual situation of patients and provide personalized suggestions when conveying knowledge and help. For example, swallowing disorders resulting from medication may be temporary and slight, so getting through the caring period may not be so challenging. For caregivers caring for patients with dysphagia due to functional deficits, education information should focus on safe eating and reducing the risk of aspiration (McCarty & Chao, 2021). While some progressive neurologic conditions, like Parkinson's disease, would permanently lose swallowing function, some patients even need to use a nasogastric tube or gastrostomy to maintain life. The group of caregivers of these patients may need more professional knowledge and continuous help.

The third theme demonstrated that family caregivers started to develop coping strategies to rebuild a normal life and gained personal growth finally after bearing the burden. The coping strategies can be mainly divided into two types of problem‐focused and emotional‐focused (Melendez et al., 2012). Problem‐focused coping relieves stress by dealing with stressors and emotional‐focused coping aims to manage emotions and feeling evoked by the source of stress (Folkman, 1984). This meta‐synthesis revealed that two main strategies are used in both, while Problem‐focused coping is used more frequently in caregivers. Caregivers can develop problem‐focused coping strategies such as acquiring knowledge related to dysphagia, seeking support, relieving the discomfort of patients and distracting attention. In contrast, the only emotional coping strategy mentioned by caregivers is getting comfort and understanding from relatives and friends. Problem‐centred coping was generally believed to improve outcomes by fostering the needed skill development and mobilizing available resources (Pires & Ugrinowitsch, 2021). At the same time, emotion‐focused coping could also assist caregivers in adapting to a stressful environment (Yu et al., 2022). A systematic review demonstrated communication and social support could increase resilient coping strategies (Palacio et al., 2020). Therefore, health workers need to keep a long‐lasting association with our caregivers and help them establish their own coping strategies.

Post‐traumatic growth perhaps offers another protective barrier against the nursing burden. Post‐traumatic growth is defined as the subjective experience of positive psychological change reported by an individual as a result of the struggle with trauma (Zoellner & Maercker, 2006). A growing appreciation of life, the feeling of increased personal strength, positive spiritual change, the setting of new life priorities and so on are the common manifestations of post‐traumatic growth (Tedeschi et al., 1998). This positive self‐change was also confirmed in our study. Dysphagia played the role of trauma. Post‐traumatic growth is a process that caregivers struggle with highly stressful events and develop a new understanding of caregiving. With the development of caring experiences, the initial passive acceptance evolved into initiative acceptance and personal strengths, for instance, feelings of responsibility and accountability, was increased in caregivers. If family members have low levels of post‐traumatic growth, they deal with the situation more negatively and passively (Perez‐San‐Gregorio et al., 2018). It may be helpful for medical staff to develop psychological interventions to promote post‐traumatic growth (Cui et al., 2017).

This meta‐synthesis identified the perceptions and experiences of caregivers of adult patients with dysphagia. Our findings provide some theoretical implications for developing supportive and family‐centered services. Overall, the results highlight the importance of comprehensive and accurate assessment of caregivers' needs, especially caregivers perceived severity of dysphagia should be assessed in routine care. Support provided by professionals could consider stress reduction strategies and increase personal pressure resistance simultaneously. It's recommended to reduce caregivers' burden by increasing medical resources, providing long‐term, individualized support and promoting family‐multidisciplinary team collaboration. Pursuing suitable countermeasures to promote caregiver personal resilience and potential, improve the post‐traumatic growth level of caregivers, and assist in developing coping strategies against stress is also imperative.

### 5.1 Limitations

There are some constraints in our study. First, we only searched the literature in standard databases and thus missed grey literature, and some articles were excluded because they were not available in full‐text form. Therefore, additional relevant studies might have been missed. Second, the methodological quality of our included research was generally moderate except for two high‐quality studies, which could potentially weaken the credibility of our findings. Moreover, only three studies in China reported economic burden, the heterogeneity among studies may exist. We assumed that might result from the difference between Chinese and Western medical and health systems or the small number of included studies; more extensive and deeper research is needed in the future to explore the sources of heterogeneity. Finally, this review was not prospectively registered.

### 5.2 Implications for future research

Additional high‐quality studies are needed to probe caring experiences in different national and/or cultural contexts. The finding points out that family caregivers experience statically significant pressure and lack of required social/healthcare support; interventions are needed to reduce the burden and promote the adaption process. Future studies should focus on exploring possible interventions and evaluating their effectiveness.

## 6 CONCLUSION

Although caregivers experienced a high caregiving burden, they respond positively to the challenge and channel the pain from distressing experiences into positive and meaningful growth. Which illustrates caring experience is complex and dynamic. Healthcare providers should pay attention to subjective perceptions, unmet needs and practical challenges of the caregivers and develop tailored interventions to reduce the burden and adjust to their new lives.

## AUTHOR CONTRIBUTIONS

QLY made substantial contributions to the conception and design of the study, researches acquisition and finished the manuscript. LYM, WYL and FS screened the articles and performed quality assessment and data extraction. LL, ZFL and HSS revised the manuscript for intellectual content. All authors have read and approved the final version of the manuscript.

## FUNDING INFORMATION

This study was funded by the 2023 Independent Innovation Fund Project from School of Nursing, Tongji Medical College, Huazhong University of Science and Technology (ZZCX2023T003).

## CONFLICT OF INTEREST STATEMENT

All the authors declare that there was no competing interest.

## RESEARCH ETHICS COMMITTEE APPROVAL

There is no requirement for ethical approval.

## Supporting information


Appendix S1.

Appendix S2.

## Data Availability

Data sharing is not applicable to this article as no new data were created or analysed in this study.
