# Supplementary material for: The perceptions and experiences of caregivers of patients with dysphagia: A qualitative meta‐synthesis
Source: Nurs Open. 2024 Sep 1;11(9):e2223. doi: 10.1002/nop2.2223 (PMC11366769; doi:10.1002/nop2.2223)
Supplement: Supplementary file 1 — Appendix S1. [file NOP2-11-e2223-s001.docx]

| **PUBMED** | | |
| --- | --- | --- |
| 1# | **Search:(((((((((dysphagia[MeSH Terms]) OR (Deglutition Disorder[Title/Abstract])) OR (Disorders, Deglutition[Title/Abstract])) OR (Swallowing Disorders[Title/Abstract])) OR (Swallowing Disorder[Title/Abstract])) OR (Dysphagia[Title/Abstract]))** | 76560 |
| 2# | **Search:((((((((caregiver*[Title/Abstract]) OR (carer*[Title/Abstract])) OR (family[Title/Abstract])) OR (spouse[Title/Abstract])) OR (relative*[Title/Abstract])) OR (parent*[Title/Abstract])) OR (kin[Title/Abstract])) OR (proxy[Title/Abstract])) OR (("Family"[Mesh]) OR "Caregivers"[Mesh])** | 3,131,685 |
| 3# | **Search:(((("Qualitative Research"[Mesh]) OR "Focus Groups"[Mesh]) OR "Grounded Theory"[Mesh]) OR "Anthropology, Cultural"[Mesh]) OR (((((((((((((((Interview[Title/Abstract]) OR (discourse[Title/Abstract])) ) OR (Phenomenol*[Title/Abstract])) OR (Content analysis[Title/Abstract])) OR (focus group[Title/Abstract])) OR (Group, Focus[Title/Abstract])) OR (Groups, Focus[Title/Abstract])) OR (Grounded Theory[Title/Abstract])) OR (Theory, Grounded[Title/Abstract])) OR (Enthnograph*[Title/Abstract])) OR (Cultural Anthropology[Title/Abstract])) OR (Material Culture[Title/Abstract])) OR (Culture, Material[Title/Abstract])) OR (Material Cultures[Title/Abstract]))** | 508,723 |
| 4# | #1 and #2 and #3 | 132 |
| **Web of science** | | |
| #1 | **(((((TS=(dysphagia)) OR TS=(Deglutition Disorder*)) OR TS=(Swallowing Disorder*)) OR AB=(dysphagia)) OR AB=(Deglutition Disorder*)) OR AB=(Swallowing Disorder*)** | 69,985 |
| #2 | **(((((((((((TS=(caregiver*)) OR TS=(family)) OR TS=(spouse)) OR TS=(carer*)) OR AB=(caregiver*)) OR AB=(family)) OR AB=(spouse)) OR AB=(carer*)) OR AB=(relative*)) OR AB=(kin)) OR AB=(parent*)) OR AB=(proxy)** | 8,928,420 |
| #3 | **((((((((((((((((AB=(Qualitative )) OR AB=( focus group)) OR AB=(Group, Focus )) OR AB=(Groups, Focus)) OR AB=( Focus Group)) OR AB=(Grounded Theory)) OR AB=(Theory, Grounded ))) OR AB=(Ethnograph*)) OR AB=(Cultural Anthropology )) OR AB=(Material Culture)) OR AB=(Culture, Material)) OR AB=(Material Cultures)) OR AB=(Interview)) OR AB=( Phenomenol*)) OR AB=(discourse)) OR AB=( Content analysis)** | 2,928,732 |
| #4 | #1 and #2 and #3 | 404 |
| **CINAHL** | | |
| S1 | AB dysphagia OR swallowing disorder* OR deglutition disorder* | 8640 |
| S2 | AB caregiver* OR family OR spouse OR carer* OR relative* OR parent* OR proxy OR kin | 591662 |
| S3 | AB Qualitative OR focus group OR Focus Group OR Group, Focus OR Groups, Focus OR Grounded Theory OR Theory, Grounded OR Ethnograph* OR Cultural Anthropology OR Material Culture OR Culture, Material OR Material Cultures OR Interview OR Phenomenol* OR discourse OR Content analysis | 319536 |
| S4 | S1 AND S2 AND S3 | 68 |
| **Ovid** | | |
| 1 | exp dysphagia/ | 150,759 |
| 2 | dysphagia.ab,ti. | 103,152 |
| 3 | Deglutition Disorder.ab,ti. | 130 |
| 4 | Disorders, Deglutition.ab,ti. | 19 |
| 5 | "Swallowing Disorder*".ab,ti. | 4,422 |
| 6 | 1 or 2 or 3 or 4 or 5 | 190,512 |
| 7 | exp caregiver/ | 110,073 |
| 8 | "caregiver*".ab,ti. | 245,165 |
| 9 | "carer*".ab,ti. | 55,103 |
| 10 | family.ab,ti. | 2,352,185 |
| 11 | spouse.ab,ti. | 29,004 |
| 12 | "relative*".ab,ti. | 4,087,145 |
| 13 | "parent*".ab,ti. | 1,255,837 |
| 14 | kin.ab,ti. | 18,164 |
| 15 | proxy.ab,ti. | 79,394 |
| 16 | 7 or 8 or 9 or 10 or 11 or 12 or 13 or 14 or 15 | 7,498,782 |
| 17 | Qualitative.ab. | 705,198 |
| 18 | focus group.ab. | 80,448 |
| 19 | Group, Focus.ab. | 365 |
| 20 | Groups, Focus.ab. | 649 |
| 21 | grounded Theory.ab. | 37,976 |
| 22 | Theory, Grounded.ab. | 214 |
| 23 | Cultural Anthropology.ab. | 386 |
| 24 | Material Culture.ab. | 899 |
| 25 | Culture, Material.ab. | 1065 |
| 26 | Interview.ab. | 383,941 |
| 27 | "Phenomenol*".ab. | 74,668 |
| 28 | discourse.ab. | 37,024 |
| 29 | Content analysis.ab. | 96,210 |
| 30 | "Ethnograph*".ab. | 30,764 |
| 31 | 17 or 18 or 19 or 20 or 21 or 22 or 23 or 24 or 25 or 26 or 27 or 28 or 29 or 30 | 1,232,405 |
| 32 | 6 and 16 and 31 | 470 |
| Cochrane Library | | |
| #1 | MeSH descriptor: [Deglutition Disorders] explode all trees | 3512 |
| #2 | (Oropharyngeal Dysphagia):ti,ab,kw OR (Dysphagia, Oropharyngeal):ti,ab,kw OR (Esophageal Dysphagia):ti,ab,kw OR (Dysphagia, Esophageal):ti,ab,kw OR (Swallowing Disorders):ti,ab,kw OR (Swallowing Disorder):ti,ab,kw OR (Disorders, Deglutition):ti,ab,kw OR (Deglutition Disorder):ti,ab,kw OR (Dysphagia):ti,ab,kw | 5672 |
| #3 | #1 OR #2 | 7855 |
| #4 | MeSH descriptor: [Caregivers] explode all trees | 3141 |
| #5 | (Family Caregiver):ti,ab,kw OR (Family Caregivers):ti,ab,kw OR (Caregiver, Family):ti,ab,kw OR (Caregivers, Family):ti,ab,kw OR (Informal Caregivers):ti,ab,kw OR (Caregivers, Informal):ti,ab,kw OR (Caregiver, Informal):ti,ab,kw OR (Informal Caregiver):ti,ab,kw OR (Spouse Caregivers):ti,ab,kw OR (Spouse Caregiver):ti,ab,kw OR (Caregivers, Spouse):ti,ab,kw OR (Caregiver, Spouse):ti,ab,kw OR (Carers):ti,ab,kw OR (Care Givers):ti,ab,kw OR (Care Giver):ti,ab,kw OR (Caregiver):ti,ab,kw OR (Carer):ti,ab,kw | 16493 |
| #6 | #4 OR #5 | 16921 |
| #7 | MeSH descriptor: [Qualitative Research] explode all trees | 1892 |
| #8 | (Research, Qualitative):ti,ab,kw | 9191 |
| #9 | #7 OR #8 | 9192 |
| #10 | #3 AND #6 AND #9 | 4 |
| ProQuest | | |
| S1 | abstract( caregiver* OR family OR spouse OR carer* OR relative* OR parent* OR proxy OR kin ) OR Exact("caregivers") | 1,028,896 |
| S2 | abstract(dysphagia OR swallowing disorder* OR deglutition disorder*) OR Exact("dysphagia") | 17,553 |
| S3 | abstract( Qualitative OR focus group OR Focus Group OR Group, Focus OR Groups, Focus OR Grounded Theory OR Theory, Grounded OR Ethnograph* OR Cultural Anthropology OR Material Culture OR Culture, Material OR Material Cultures OR Interview OR Phenomenol* OR discourse OR Content analysis ) | 379,691 |
| S4 | S1 AND S2 AND S3 | 105 |
| CNKI | | |
| 1 | TKA(title OR keywords OR abstract)=('dysphagia'+' swallowing disorder ')*('caregiver'+'family'+'relative*')*('qualitative'+'focus group'+' Grounded Theory *') | 12 |
| WanFang | | |
| 2 | Title or Keywords:(" dysphagia "or " swallowing disorder ") AND ("caregiver"or"family"or"relative*") AND ("qualitative research"or"focus group"or" Grounded Theory ") | 3 |
